# Histochemical mapping of the duration of action of photobiomodulation on cytochrome c oxidase in the rat brain

**DOI:** 10.3389/fnins.2023.1243527

**Published:** 2023-08-28

**Authors:** Zachary S. Wade, Douglas W. Barrett, Roger E. Davis, Adrian Nguyen, Sindhu Venkat, F. Gonzalez-Lima

**Affiliations:** Department of Psychology and Institute for Neuroscience, The University of Texas at Austin, Austin, TX, United States

**Keywords:** *in vivo* photobiomodulation, transcranial infrared laser stimulation, brain cytochrome c oxidase, hormesis, prefrontal cortex, neuroenergetics, enzyme histochemistry, low-level laser therapy

## Abstract

**Introduction:**

This is the first study mapping the duration of action of *in vivo* photobiomodulation (PBM) on cytochrome-c-oxidase (CCO). In cellular bioenergetics, CCO is the terminal rate-limiting enzyme in the mitochondrial electron transport chain, which catalyzes oxygen utilization for aerobic energy production. PBM using transcranial infrared laser stimulation (TILS) is a promising intervention for non-invasively modulating CCO in the brain. TILS of the human prefrontal cortex directly causes CCO photo-oxidation, which is associated with increased cerebral oxygenation and improved cognition.

**Methods:**

This experiment aimed to map the duration of action of *in vivo* PBM on CCO activity in discrete neuroanatomic locations within rat brains up to 4 weeks after a single TILS session (50 s, 1064 nm CW, 250 mW/cm^2^). Control brains from rats treated with a sham session without TILS (laser off) were compared to brains from TILS-treated rats that were collected 1 day, 2 weeks, or 4 weeks post-TILS. Cryostat sections of the 36 collected brains were processed using quantitative enzyme histochemistry and digitally imaged. Densitometric readings of 28 regions of interest were recorded and converted to CCO activity units of oxygen utilization using calibration standards. Data analysis (ANCOVA) compared each laser-treated group to sham with whole-brain average as a covariate.

**Results:**

The prefrontal infralimbic cortex showed the earliest significant increase in CCO activity between 1-day post-TILS and sham groups, which continued elevated for 2–4 weeks post-TILS. Significant differences in CCO activity between 2-weeks and sham groups were also found in the lateral septum, accumbens core, CA3 of the hippocampus, and the molecular layer of the hippocampus. The medial amygdala showed a significant decrease in CCO activity between 4-weeks and sham. Further analyses showed significant inter-regional CCO activity correlations among the brain regions as the result of TILS, with the most pronounced changes at 4-weeks post-stimulation.

**Discussion:**

The time course of changes in CCO activity and network connectivity suggested that TILS caused different neuroplasticity types of bioenergetic changes at different time scales, depending on brain region and its depth from the cortex. In conclusion, this controlled CCO histochemical study demonstrated a long-lasting duration of action of PBM in the rat brain.

## Introduction

Cytochrome-c-oxidase (CCO) is a mitochondrial enzyme that reduces oxygen to water in the electron transport chain. This leads to increased oxidative phosphorylation of adenosine triphosphate (ATP) that promotes aerobic bioenergetic activity (Hatefi, 1985). Cognitive performance is associated with aerobic bioenergetic activity in both animal and human studies (Messier, 2004; Hatchard et al., 2014). Neuronal bioenergetic activity is highly dependent on the activity of CCO (Wong-Riley, 1989). CCO is the major intracellular acceptor of light in the red to near-infrared wavelengths (Karu, 1999; Rojas and Gonzalez-Lima, 2011). In rat models, photobiomodulation (PBM; Anders et al., 2015) has been shown to improve oxygenation of the prefrontal cortex (Rojas et al., 2012) as well as the whole brain (Rojas et al., 2008). PBM increases ATP synthesis by mitochondria, promoting an increase in energy metabolism (Rojas and Gonzalez-Lima, 2013; Dole et al., 2023) and modifying multiple metabolic pathways revealed by molecular metabolomics of the rat brain (Dos Santos Cardoso et al., 2021).

Transcranial infrared laser stimulation (TILS) is a type of *in vivo* PBM that involves the application of directional low-power and high-fluence monochromatic 1,064 nm infrared light to the surface of the head. A portion of this light penetrates the skull and is absorbed by the chromophores in CCO, causing a direct photonic oxidation of CCO that is not mediated by a thermal effect (Wang et al., 2022). For example, four recent sham-controlled studies have confirmed that *in vivo* oxidation of CCO is a direct photonic action of TILS administration to the human prefrontal cortex (Wang et al., 2017, 2018; Pruitt et al., 2020; Saucedo et al., 2021). This supports the utility of a PBM method like TILS, which directly oxidizes CCO, in modifying brain bioenergetic capacity.

While many studies have investigated the ability of TILS to modulate CCO in the brain, the temporal duration of TILS effects on neural CCO activity is currently unknown. Studies on the effects of a single session of TILS in human subjects have revealed enhancement of cognitive function in the domains of processing speed, sustained attention, executive function, working memory and category learning (Barrett and Gonzalez-Lima, 2013; Gonzalez-Lima and Barrett, 2014; Blanco et al., 2015, 2017). Notably, multiple weeks of TILS treatment in human subjects have further shown improvements in reaction time and working memory (Vargas et al., 2017). In aged rat models, repeated TILS treatments produced beneficial brain bioenergetic effects by reversing the effects of aging on CCO activity (Cardoso et al., 2022). Prior studies that examine the behavioral and neurophysiological effects of TILS have focused on the time period during or immediately following TILS treatment (Holmes et al., 2019; Wang et al., 2019). Because of this, the duration of action of a single administration of TILS is not known.

This study aims to histochemically quantify the duration of rat brain CCO activity in cortical and subcortical regions of interest (ROIs) over 4 weeks following a single TILS administration. Based on published findings, we hypothesized that rats in groups that receive TILS would show modified levels of CCO activity when compared to rats that receive a sham procedure (Rojas et al., 2012). We further hypothesized that CCO activity changes in key ROIs such as the prefrontal cortex would be found 1 day after stimulation, with longer-lasting changes in CCO activity at 2 and 4 weeks after TILS.

## Materials and methods

### Animals

The subjects of the present study were 47 male Sprague–Dawley rats (sham *n* = 9, TILS-treated *n* = 27, homogenate paste *n* = 10, light distribution *n* = 1). Male rats were chosen to preclude the need for estrous cycle testing. An albino strain was used to eliminate pigmentation as a potential barrier to transcranial infrared light penetration. All rats were 8 weeks old and weighed an average of 250 grams at the time of TILS or sham administration. Food was provided *ad libitum* throughout the experimental period. The animals were pair-housed at a temperature of 21 ± 2°C with a 12 h light/dark cycle. All procedures were approved by the Institutional Animal Care and Use Committee at the University of Texas at Austin. Frozen brain tissue was processed and analyzed at the University of Texas at Austin, following all institutional laboratory safety guidelines.

### Laser treatment

**Figure 1:**
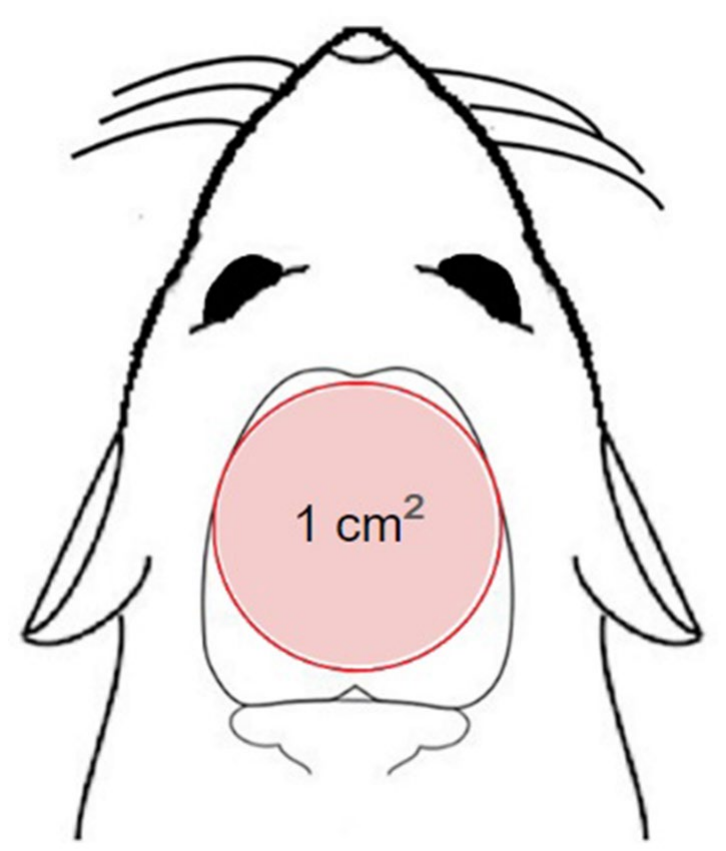
Diagram of laser irradiation point on the scalp centered between the ears.

Beginning 1 week before the TILS protocol, all subjects were handled daily to habituate them to the one-minute immobilization needed for laser stimulation. Each rat was assigned to one of three cohorts consisting of 12 rats. Three rats in each cohort were randomly assigned to the sham control condition; the remaining nine rats in each cohort were divided into three treatment groups, which determined when they were decapitated after treatment. All rats were manually immobilized without anesthesia for 1 min, during which the rats in the treatment groups received 50 s of stimulation from a laser diode with a measured power output of 3.4 W, an irradiance of 250 mW/cm^2^, a wavelength of 1,064 nm, and an energy density of 12.5 J/cm^2^. We used a continuous wave (CW) laser, and the irradiance (250 mW/cm^2^) was the top-hat laser output measured at the head surface with a photometer (Newport model 1919-R power meter, Newport model 918D-SL photodiode detector). The same laser device, wavelength and irradiance used for our TILS studies in humans was used in the rats (HD Laser, Cell Gen Therapeutics, Dallas, Texas, USA). But a shorter exposure time period when compared to human subjects was warranted due to relatively small size of the rat brain as well as the thinner bones of the rat skull. Both of these factors increased the quantity of penetrating light from the TILS treatment. The laser diode was positioned posterior to the rats’ eyes to avoid unintentional eye damage. We chose a single irradiation point centered at the interaural line on the top of the rat head with an area of 1 cm^2^ (Figure 1) to target a majority of the brain. Rats in the sham control group did not receive laser stimulation, but were handled and restrained in the same way and for the same one-minute duration as the rats in the TILS treatment groups. All rats in the sham control group and the one-day group were decapitated 1 day after the sham procedure/TILS treatment. All rats in the 2-weeks group were decapitated 2 weeks after TILS treatment, and all rats in the four-weeks group were decapitated 4 weeks after TILS treatment. After decapitation, all brains were quickly extracted and immediately frozen for subsequent cryostat sectioning.

### Quantitative histochemistry of brain cytochrome c oxidase

Coronal sections of the brain (40 μm thick) were obtained using a Reichert-Jung cryostat at −20°C and processed for subsequent CCO enzyme histochemistry, following the quantitative method previously described in detail by Gonzalez-Lima and Cada (1998). All brains were processed in this way, with the exception of one brain from the four-week post-TILS decapitation group, which had significant tissue damage due to desiccation while in frozen storage. Calibration slides were prepared with frozen rat brain paste homogenate sectioned into 10, 20, 40, 60, and 80 μm thick sections. Spectrophotometry of each paste homogenate served to quantify calibration standards of CCO activity units. Slides with these quantified brain paste homogenate sections were included with each CCO staining batch as calibration standards. The slides were fixed for 5 min with 0.5% vol/vol glutaraldehyde and then were rinsed three times in 0.1 M phosphate buffer with 10% wt/vol sucrose (pH 7.6). Slides were pre-incubated in a solution containing 275 mg/L cobalt chloride, 10% wt/vol sucrose and 0.5% vol/vol dimethyl sulfoxide dissolved in Tris buffer (pH 7.6). For histochemical staining, slides were then incubated in the dark at 37°C for 1 h in a continuously-stirred solution containing 350 mg diaminobenzidine tetrahydrochloride, 35 g sucrose, 52.5 mg cytochrome c and 14 mg catalase dissolved in 700 ml of oxygen-saturated 0.1 M phosphate buffer (pH 7.6). Immediately after incubation, the slides were dehydrated in a series of ethanol baths increasing from 30 to 100% vol/vol ethanol. Slides were then cleaned with xylene, and finally coverslips were applied using Permount.

**Figure 2:**
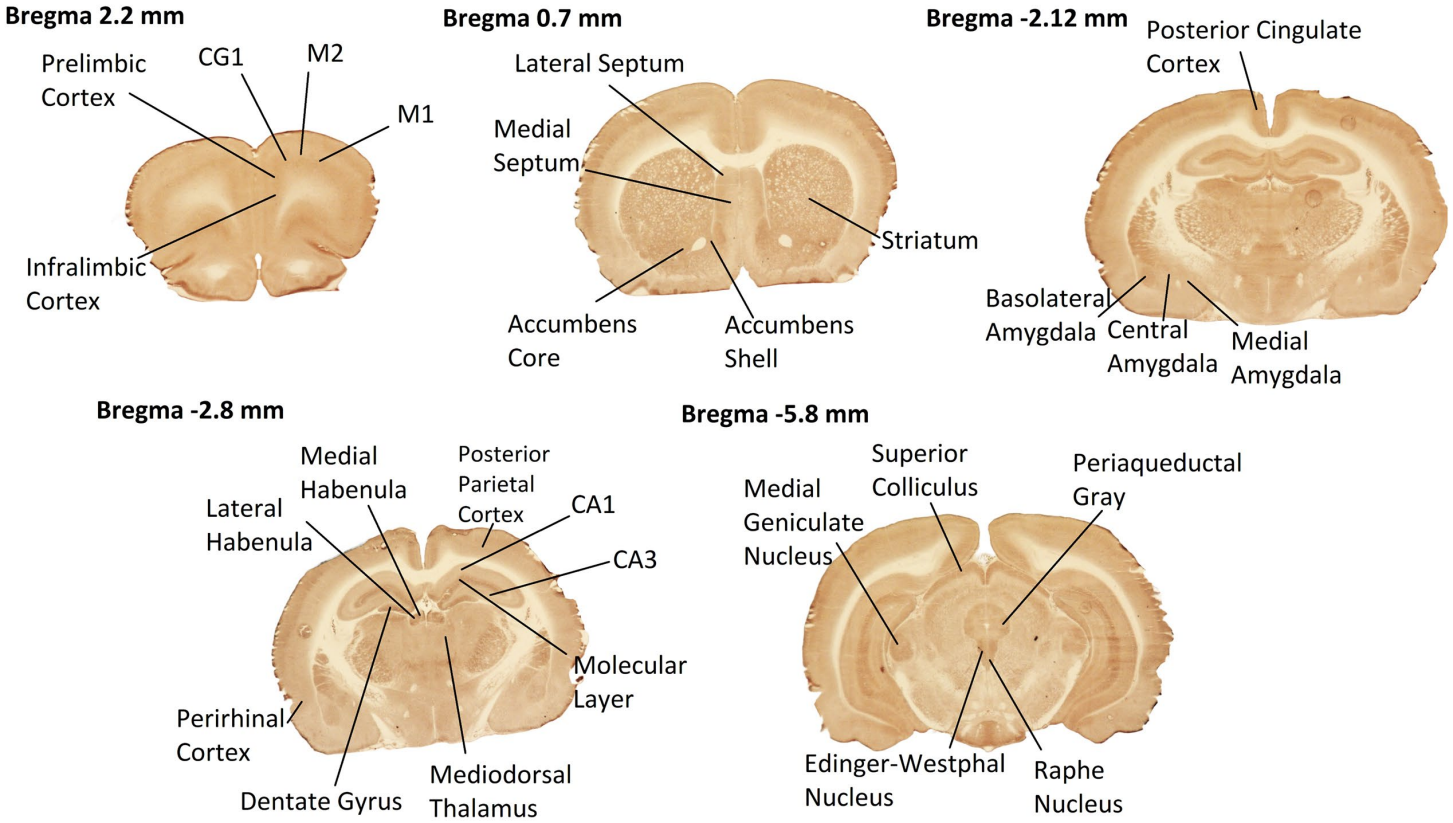
Rat brain ROIs from CCO-stained sections used in this study. Coronal sections at 5 Bregma levels distributed throughout the entire rat brain were histochemically stained for CCO enzymatic activity. Darker staining means greater CCO activity. Neuroanatomic ROIs are labeled. Anterior–posterior coordinates from the Bregma landmark are noted at the top of each of the images. Selected sections exhibited high quality of tissue integrity after sectioning, staining, and image capture.

High-resolution images of each section were captured using a high-precision, stable, and uniform illuminator, and digital camera (Cardoso et al., 2022). The density of CCO histochemical staining of these brain section images were analyzed with the image analysis software ImageJ. Optical density (OD) readings were obtained from 28 brain ROIs (Figure 2) as defined in the rat brain atlas of Paxinos and Watson (2006): primary motor cortex (M1), secondary motor cortex (M2), cingulate cortex (CG1), prelimbic cortex, and infralimbic cortex at Bregma 2.2 mm; striatum, lateral septum (LS), medial septum (MS), accumbens shell, and accumbens core at Bregma 0.7 mm; posterior cingulate cortex, basolateral amygdala (AB), central amygdala (ACe), and medial amygdala (AMe) at Bregma −2.12 mm; field CA1 of the hippocampus, field CA3 of the hippocampus, molecular layer of the hippocampus, dentate gyrus (DG), posterior parietal cortex, perirhinal cortex (PRh), mediodorsal nucleus of the thalamus (MD), lateral habenula (LHb), and medial habenula (MHb) at Bregma −2.80 mm; periaqueductal gray (PAG), superior colliculus, medial geniculate nucleus (MG), Edinger-Westphal nucleus, and raphe nucleus at Bregma −5.80 mm. These ROIs were chosen to include a robust distribution of both anterior-to-posterior coordinates and cortical vs. subcortical regions throughout the entire rat brain.

### Correlation between histochemical staining and fresh brain tissue CCO activity measured biochemically

For the biochemical assay, we used fresh unfixed brain homogenate paste from 10 additional rats and measured CCO activity using spectrophotometry, as described in detail before (Gonzalez-Lima and Cada, 1998). We made 10 batches of CCO staining that were correlated with the biochemical assay values. Each batch had 24 tissue sections with the spectrophotometrically measured CCO activity. The CCO activity correlation between the 240 fresh tissue samples and histochemically stained sections in the 10 batches yielded a mean regression coefficient of 0.97 (standard deviation 0.014, minimum 0.95, maximum 0.99). This served to confirm that the CCO activity measured by enzyme histochemistry after a 5-min fixation step with 0.5% glutaraldehyde was highly correlated with CCO activity measured by spectrophotometry in fresh unfixed tissue, as previously demonstrated (Gonzalez-Lima and Cada, 1998).

### Statistical analysis

To account for potential variances in staining between brain sections of different staining batches, mean optical density (OD) of CCO-stained brain homogenate paste standards were recorded. Regression calibration curves were calculated for each batch between each homogenate brain paste standard section thickness and its spectrophotometrically-measured CCO enzymatic activity level. The mean OD measured histochemically in each ROI was converted to CCO enzymatic activity units (μmol of cytochrome c oxidized/min/g of wet tissue weight) using the regression curve calculated for each batch. Descriptive statistics were then calculated. All statistical analyses were performed using the jamovi open access statistical analysis software (The jamovi project, 2022). Statistical significance was set at *p* < 0.05, two-tailed for all comparisons.

A series of analyses of covariance (ANCOVAs) compared CCO activity between treatment and sham groups in each brain ROI, using the whole brain average as a covariate. For each ROI, three ANCOVAs were performed, comparing 1-day to sham, 2-weeks to sham, and 4-weeks to sham. By averaging the CCO activity of all regions within each Bregma level, analyses of variance (ANOVAs) were used to examine group effects by Bregma level. Partial correlations of CCO activity between each ROI were computed for each group, controlling for whole brain mean, as previously described (Cardoso et al., 2022). The number of significant partial correlations in each post-TILS group was compared to the number of significant partial correlations in the sham group.

Almost the entire dorsal surface of the rat brain was irradiated with light during TILS treatment. Amount of light absorbed from TILS varies between ROIs with differences in distance from the site of stimulation. To assess for any differential TILS effects on cortical and subcortical regions of the brain, each brain ROI was categorized as either cortical or subcortical. Separate ANOVAs were conducted to evaluate any significant group mean differences between cortical and subcortical regions. Brain regions of interest were categorized by the approximate dorsoventral depth of optical density readings, measured from the dorsal surface of the cortex (Paxinos and Watson, 2006). Each ROI was categorized into one of 7 groups based on dorsoventral depth. Linear regression analysis with group and dorsoventral depth as factors assessed for any significant association between depth of brain ROIs and CCO activity. Dorsoventral depth and categorization of each brain ROI are presented in Table 1.

**Table 1:** Categorization of each brain ROI by cortical or subcortical location and dorsoventral depth.

	Cortical or subcortical	Dorsoventral depth	Depth category
M1	Cortical	1.2 mm	1–1.9 mm
M2	Cortical	1 mm	1–1.9 mm
CG1	Cortical	2 mm	2–2.9 mm
Prelimbic	Cortical	3.2 mm	3–3.9 mm
Infralimbic	Cortical	4.3 mm	4–4.9 mm
Striatum	Subcortical	5 mm	5–5.9 mm
LS	Subcortical	4.6 mm	4–4.9 mm
MS	Subcortical	5.4 mm	5–5.9 mm
Accumbens shell	Subcortical	6.8 mm	6–6.9 mm
Accumbens core	Subcortical	6.9 mm	6–6.9 mm
Posterior cingulate	Cortical	1.4 mm	1–1.9 mm
AB	Subcortical	7.8 mm	7–8.2 mm
ACe	Subcortical	7.4 mm	7–8.2 mm
AMe	Subcortical	8.2 mm	7–8.2 mm
CA1	Subcortical	2.2 mm	2–2.9 mm
CA3	Subcortical	3 mm	3–3.9 mm
Molecular layer	Subcortical	2.8 mm	2–2.9 mm
DG	Subcortical	3.4 mm	3–3.9 mm
Posterior parietal	Cortical	1 mm	1–1.9 mm
PRh	Cortical	6.8 mm	6–6.9 mm
MD	Subcortical	5.2 mm	5–5.9 mm
LHb	Subcortical	4.8 mm	4–4.9 mm
MHb	Subcortical	4.7 mm	4–4.9 mm
PAG	Subcortical	5 mm	5–5.9 mm
Superior colliculus	Subcortical	3.2 mm	3–3.9 mm
MG	Subcortical	5.2 mm	5–5.9 mm
Edinger Westphal	Subcortical	6 mm	6–6.9 mm
Raphe	Subcortical	6.2 mm	6–6.9 mm

Frequency of ROIs in each cortical or subcortical category: cortical (*n* = 8), subcortical (*n* = 20). Frequency of ROIs in each depth category: 1–1.9 mm (*n* = 4), 2–2.9 mm (*n* = 3), 3–3.9 mm (*n* = 4), 4–4.9 mm (*n* = 4), 5–5.9 mm (*n* = 5), 6–6.9 mm (*n* = 5), 7–8.2 mm (*n* = 3).

## Results

### Whole brain CCO activity in the TILS groups versus sham

There were no significant differences in the whole brain CCO activity in the TILS groups versus the sham. Whole brain means of CCO activity units showed a small trend to increase as a function of time after TILS: group 1 (sham) = 164, group 2 (1-day) = 165, group 3 (2-weeks) = 179, group 4 (4-weeks) = 186. However, an ANOVA between the four groups did not show significant whole brain mean group differences (*p* = 0.277). Similarly, individual group comparisons with ANOVA did not show any significant differences: group 1 vs. 2 (*p* = 0.873), group 1 vs. 3 (*p* = 0.301), group 1 vs. 4 (*p* = 0.200).

### Duration of action of TILS on regional mean CCO activity

**Figure 3:**
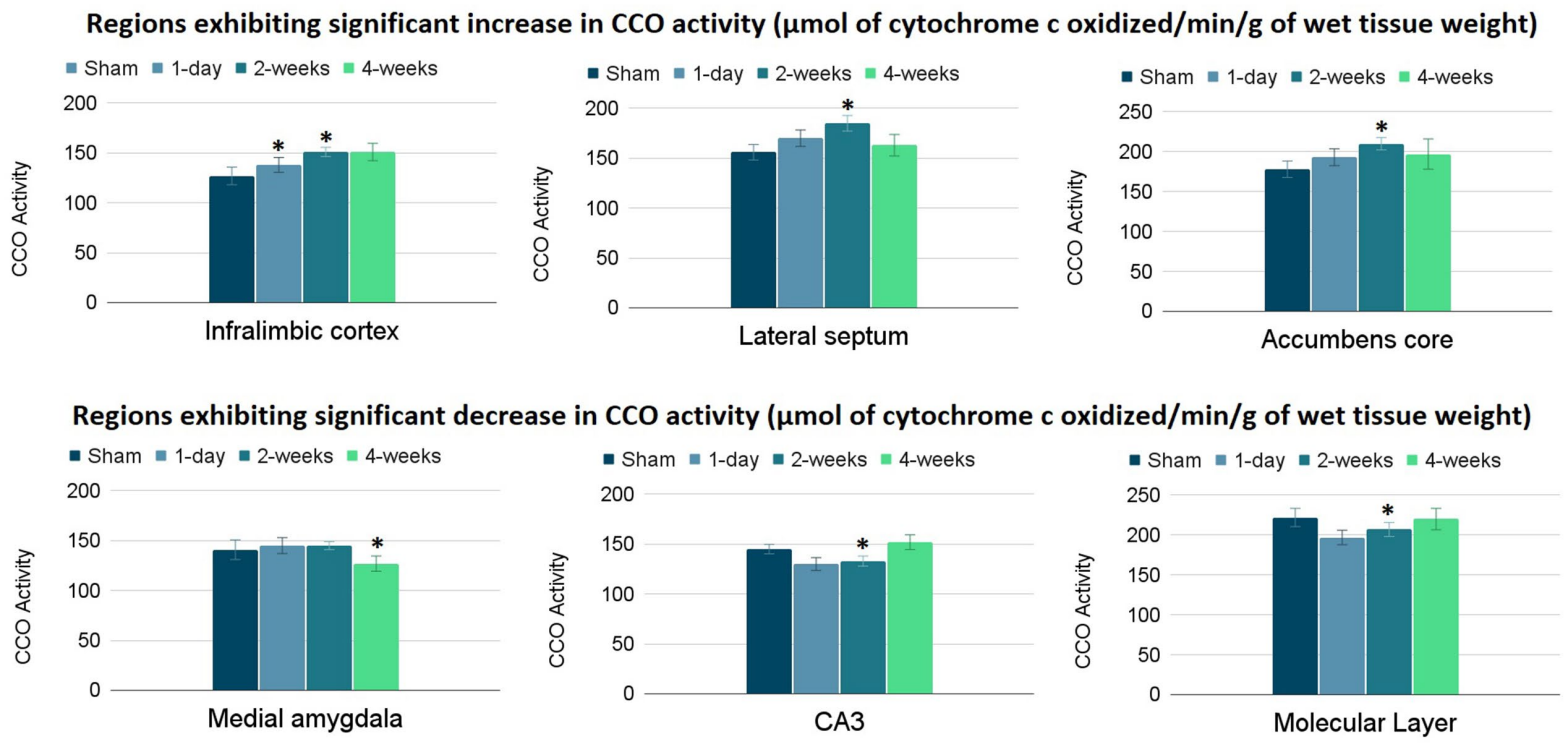
Significant changes in CCO activity (μmol of cytochrome c oxidized/min/g of wet tissue weight) of 6 ROIs post-TILS treatment. Top row: significant increases; bottom row: significant decreases. CCO activity increases were noted in the infralimbic cortex of the 1-day post-TILS group compared to sham and from the 2-weeks post-TILS group to sham. Significant increases in CCO activity were also recorded in the lateral septum and accumbens core of the 2-weeks post-TILS group compared to sham. Decreases in CCO activity were recorded in the medial amygdala of the 4-weeks post-TILS group compared to sham. Significant decreases in CCO activity were also recorded in the CA3 and the molecular layer of the hippocampus of the 2-weeks post-TILS group compared to sham. * indicates a *p*-value < 0.05.

The group means and standard errors for each brain ROI are presented in Table 2. Three ANCOVAs were conducted to compare regional mean CCO activity between each post-TILS group to the sham group for all ROIs, using the whole brain average as a covariate. Regions showing significant elevation in CCO activity are visualized in the top row of Figure 3, and regions showing significant reduction in CCO activity are visualized in the bottom row of Figure 3. In an ANCOVA comparing the 1-day post-TILS group to the sham group, the prefrontal infralimbic cortex showed a significant mean increase in CCO activity in the 1-day post-TILS group compared to sham (*p* = 0.04). The infralimbic cortex also showed a significant mean increase in CCO activity in the 2-weeks post-TILS group compared to sham (*p* = 0.023). Other ROIs that displayed a significant increase in CCO activity in the 2-weeks post-TILS group compared to the sham group included the LS (*p* = 0.015) and the accumbens core (*p* = 0.017). CCO activity in both CA3 (p = 0.02), and the molecular layer of the hippocampus (*p* = 0.009) were significantly reduced in the 2-weeks post-TILS group compared to sham. When comparing CCO activity in the four-weeks post-TILS group to sham, the AMe was the only ROI to demonstrate a significant change from the sham (*p* = 0.036) with a significant decrease in CCO activity.

**Table 2:** Means and standard errors of cytochrome c oxidase activity (μmol of cytochrome c oxidized/min/g of wet tissue weight) of all ROIs from sham, 1-day, 2-weeks, and 4-weeks groups.

Mean ± SE				
	Sham (*n* = 9)	1-day (*n* = 9)	2-weeks (*n* = 9)	4-weeks (*n* = 8)
M1	149 ± 12.6	147 ± 11.8	159 ± 8.51	154 ± 7.32
M2	144 ± 12.8	140 ± 8.36	156 ± 8.45	141 ± 11.0
CG1	145 ± 12.4	145 ± 7.59	152 ± 4.03	159 ± 8.80
Prelimbic	139 ± 9.86	144 ± 7.01	161 ± 7.96	159 ± 7.53
Infralimbic	127 ± 8.90	**138 ± 7.50**	**151 ± 4.58**	151 ± 8.69
Striatum	183 ± 4.74	187 ± 7.81	206 ± 11.5	189 ± 17.1
LS	156 ± 7.82	170 ± 8.20	**185 ± 7.88**	163 ± 10.8
MS	123 ± 4.86	120 ± 7.95	150 ± 11.1	123 ± 7.66
Accumbens shell	169 ± 11.1	187 ± 12.2	193 ± 8.94	175 ± 12.5
Accumbens core	178 ± 10.3	193 ± 10.5	**210 ± 7.68**	197 ± 19.0
Posterior cingulate	202 ± 13.4	190 ± 7.98	210 ± 6.84	205 ± 11.3
AB	159 ± 12.6	168 ± 4.97	166 ± 8.14	186 ± 9.56
ACe	143 ± 9.51	141 ± 5.29	146 ± 7.12	158 ± 9.89
AMe	141 ± 9.86	145 ± 7.94	145 ± 4.02	**127 ± 7.63**
CA1	120 ± 6.27	121 ± 5.98	115 ± 4.67	131 ± 6.82
CA3	145 ± 4.74	130 ± 6.47	**133 ± 5.05**	152 ± 7.35
Molecular layer	222 ± 11.4	197 ± 9.12	**207 ± 8.76**	220 ± 13.5
DG	213 ± 10.2	191 ± 11.1	206 ± 8.46	236 ± 9.80
Posterior parietal	155 ± 4.89	150 ± 7.46	149 ± 8.79	163 ± 10.3
PRh	152 ± 7.71	148 ± 10.2	141 ± 5.94	157 ± 11.1
MD	157 ± 10.9	156 ± 8.29	148 ± 6.70	160 ± 6.10
LHb	182 ± 10.1	178 ± 15.0	191 ± 6.54	207 ± 10.3
MHb	207 ± 11.0	198 ± 14.5	222 ± 15.9	230 ± 10.6
PAG	167 ± 12.1	159 ± 6.51	193 ± 17.8	219 ± 22.3
Superior colliculus	204 ± 13.9	193 ± 5.79	226 ± 22.5	259 ± 25.5
MG	181 ± 11.4	174 ± 6.74	212 ± 17.6	216 ± 13.7
Edinger Westphal	198 ± 12.7	184 ± 15.1	210 ± 29.4	249 ± 23.7
Raphe	241 ± 17.9	219 ± 9.40	256 ± 33.9	305 ± 25.2

Bold values denote ROIs with *p* value ≤ 0.05 compared to sham in ANCOVA, using the whole brain average as a covariate.

An ANOVA comparing the between-group means of each Bregma level revealed a significant effect of group only in Bregma −5.8 (*p* = 0.029). ANOVAs that were conducted to assess for possible effects of group on cortical and subcortical CCO activity means revealed no significant effects, with a cortical *p*-value of 0.430 and a subcortical *p*-value of 0.329.

### Duration of action of TILS on inter-regional CCO activity correlations

**Figure 4:**
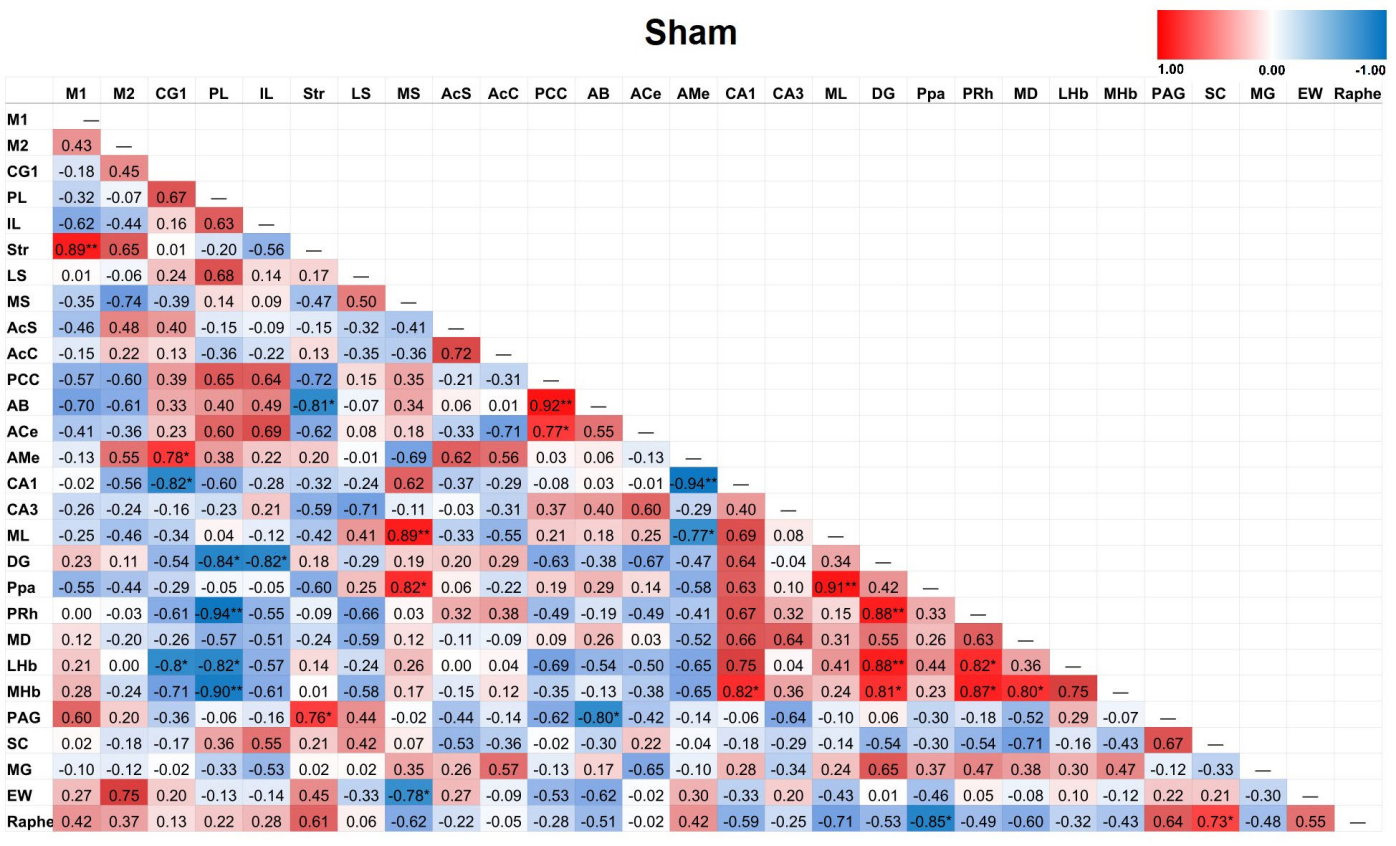
Heat color map of Pearson’s r values for each pairwise correlation between two ROIs in the sham group. Red indicates positive inter-regional correlations and blue indicates negative correlations. * indicates a *p*-value < 0.05. ** indicates a *p*-value < 0.01. *** indicates a *p*-value < 0.001.

**Figure 5:**
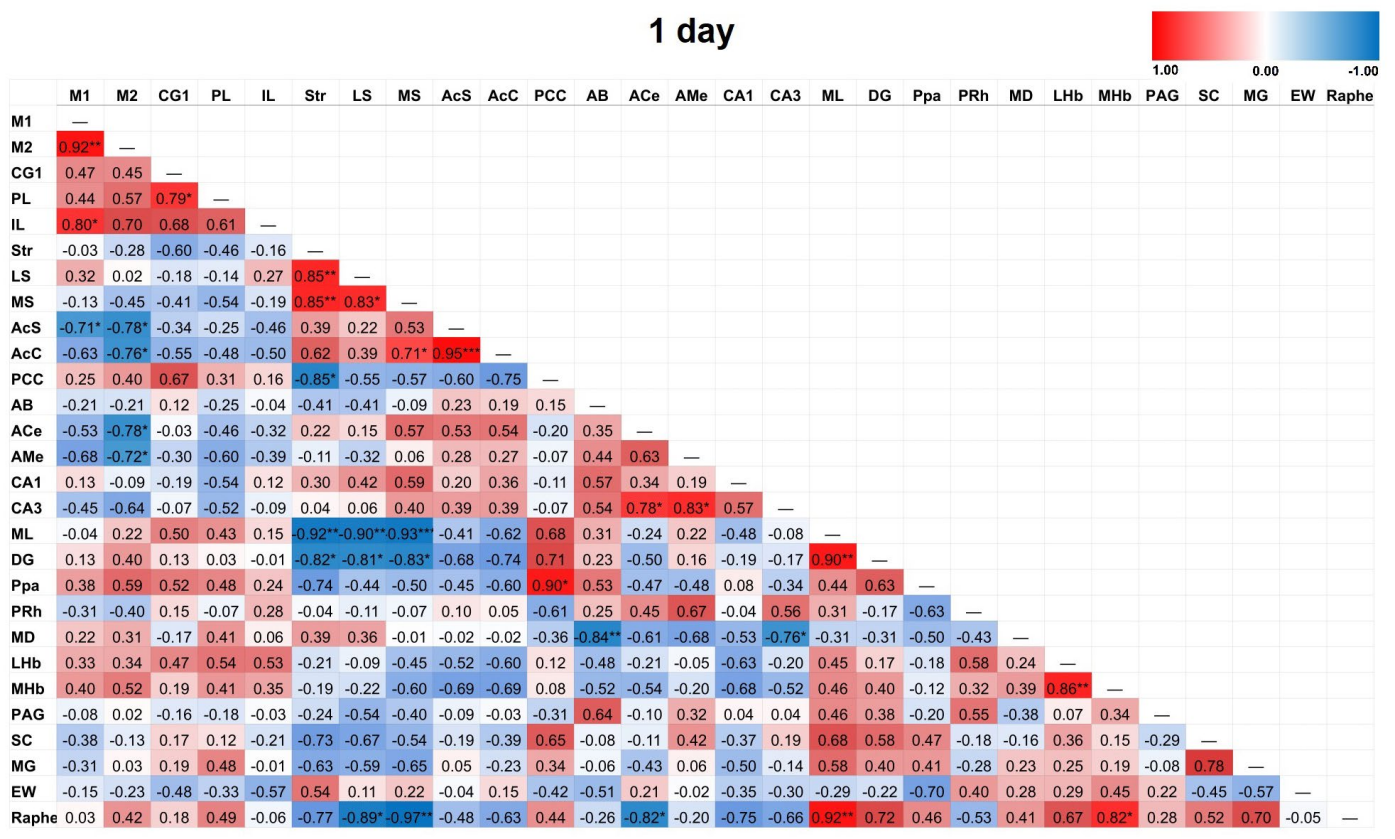
Heat color map of Pearson’s r values for each pairwise correlation between two ROIs in the 1-day group. Red indicates positive inter-regional correlations and blue indicates negative correlations. * indicates a *p*-value <0.05. ** indicates a *p*-value < 0.01. *** indicates a *p*-value < 0.001. At 1-day post-TILS, there were 31 additional significant correlations that were not found in sham. Increased number of significant partial correlations over time post-stimulation suggests increased functional connectivity between brain ROIs.

**Figure 6:**
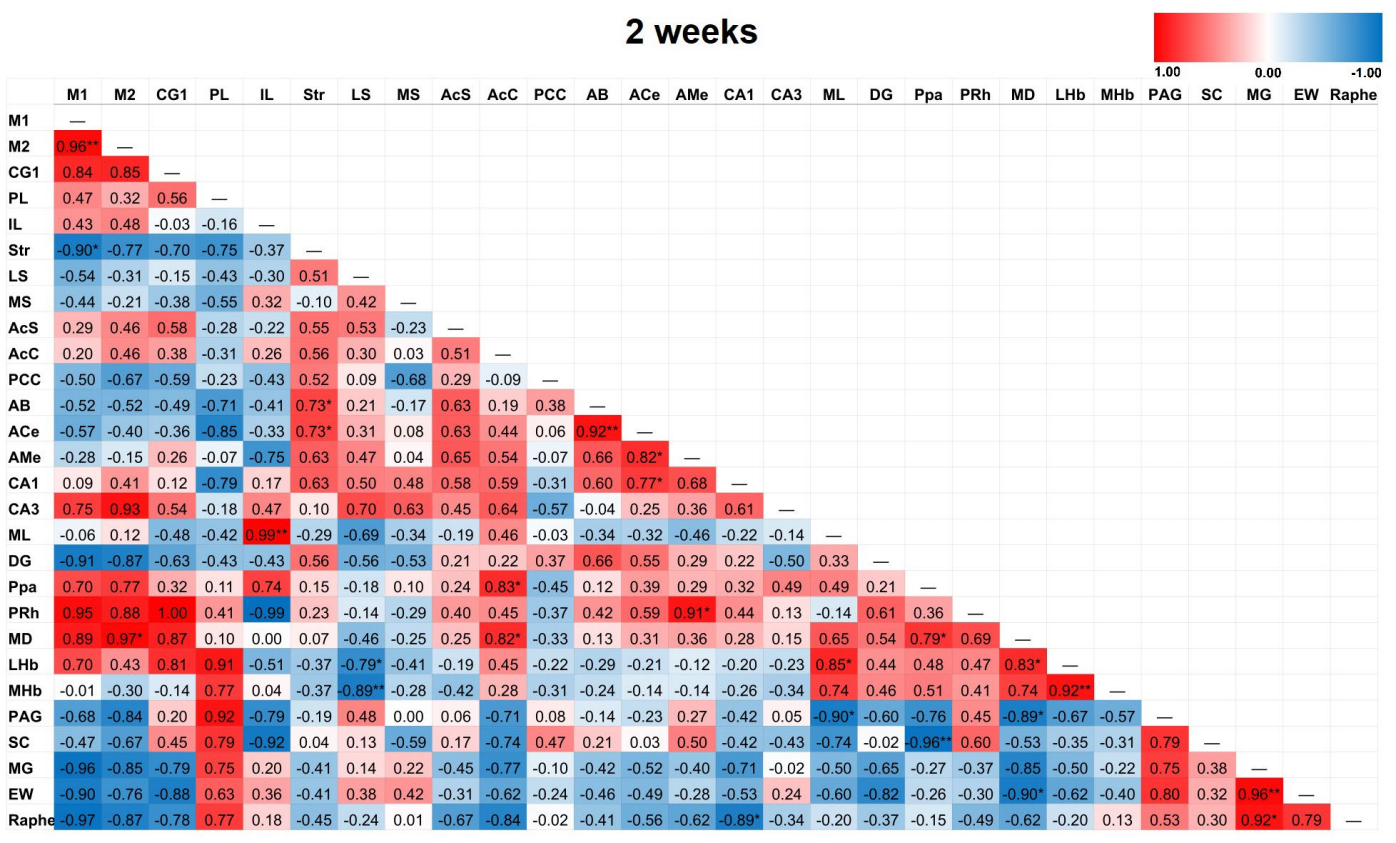
Heat color map of Pearson’s r values for each pairwise correlation between two ROIs in the 2-week group. Red indicates positive inter-regional correlations and blue indicates negative correlations. * indicates a *p*-value < 0.05. ** indicates a *p*-value < 0.01. *** indicates a *p*-value < 0.001. At 2-weeks post-TILS, there were 23 additional significant correlations that were not found in sham. Increased number of significant partial correlations over time post-stimulation suggests increased functional connectivity between brain ROIs.

**Figure 7:**
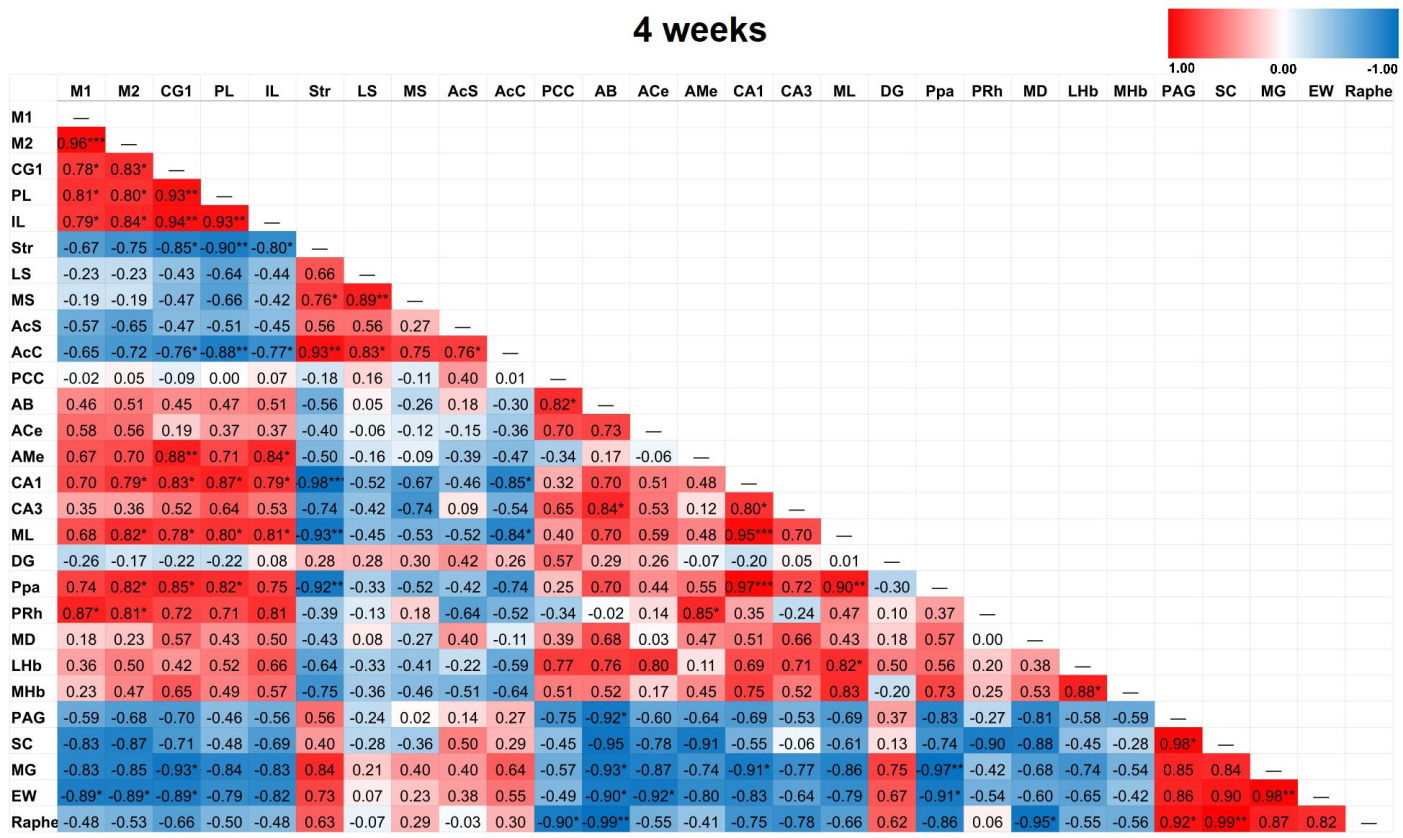
Heat color map of Pearson’s r values for each pairwise correlation between two ROIs in the 4-week group. Red indicates positive inter-regional correlations and blue indicates negative correlations. * indicates a *p*-value < 0.05. ** indicates a *p*-value < 0.01. *** indicates a *p*-value < 0.001. At 4-weeks post-TILS, there were 61 additional significant correlations that were not found in sham. Increased number of significant partial correlations over time post-stimulation suggests increased functional connectivity between brain ROIs.

Partial correlation matrices for the three post-TILS groups compared to sham assessed for any changes in the overall number of significant partial pairwise correlations between ROIs. Whole brain average was used as a covariate (Cardoso et al., 2022). Figures 4–7 show heat color maps from each treatment condition, displaying Pearson’s r values for each pairwise correlation between two ROIs. The red color indicates the presence of positive correlations, which increased as function of time after TILS (visualized as more red colors from Figures 4 through 7). Partial correlation analysis revealed 27 significant partial pairwise correlations between brain ROIs in the sham condition. When compared to sham, the 1-day post-TILS group displayed 31 significant pairwise partial correlations between ROIs that were not significant in the sham group. The 2-weeks post-TILS group displayed 23 significant pairwise partial correlations between ROIs that were not significant in the sham group. Finally, the 4-weeks post-TILS group exhibited 61 significant pairwise partial correlations between ROIs that were not significant in the sham group.

### Association between TILS and CCO activity depending on brain depth

**Figure 8:**
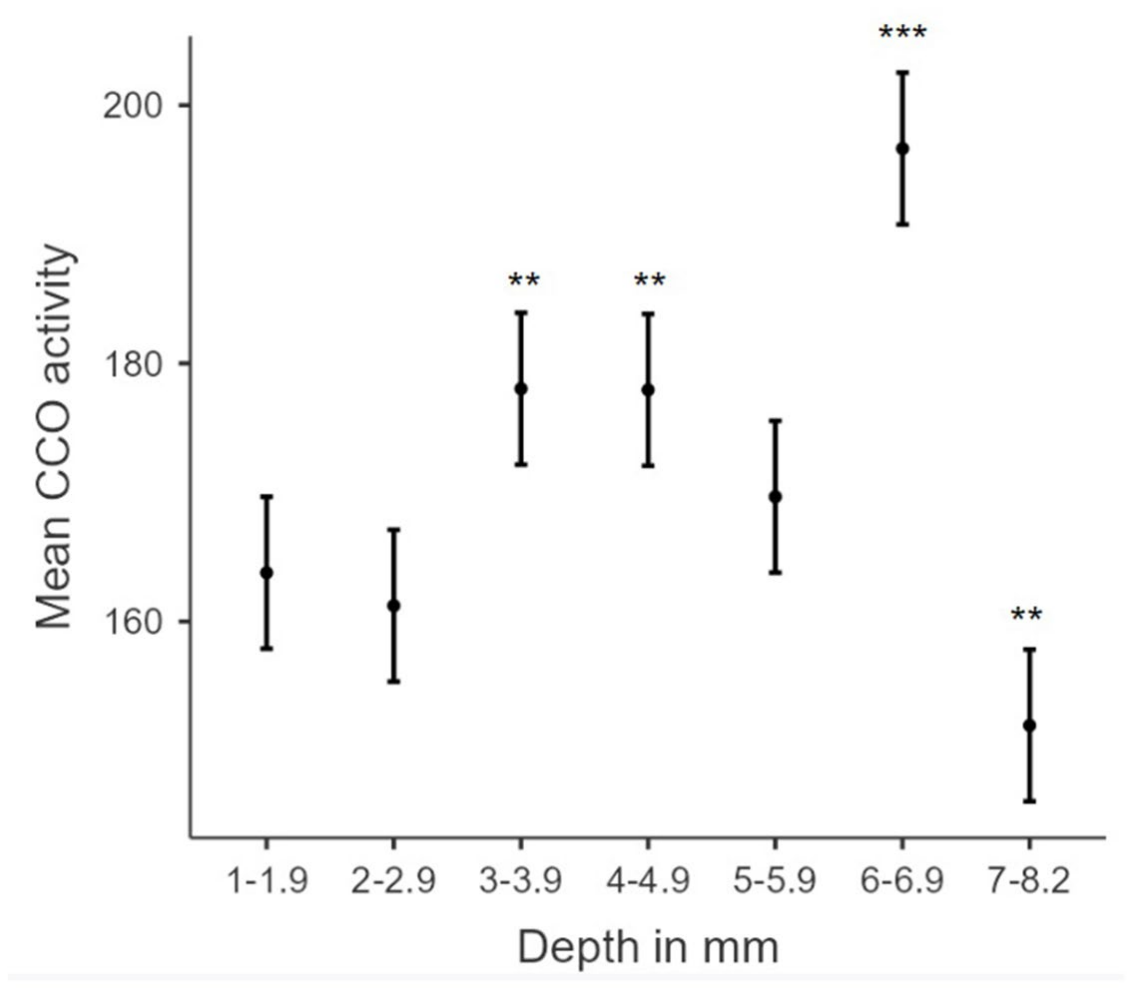
Biphasic hormetic trend in mean CCO activity: increasing CCO activity with increasing dorsoventral depths 1–6.9 mm, and decreasing CCO activity in dorsoventral depths greater than 7 mm. Plot of estimated marginal means for dorsoventral depth from the dorsal surface of the brain (mm) from the linear regression versus mean cytochrome c oxidase activity (μmol of cytochrome c oxidized/min/g of wet tissue weight). * indicates a *p*-value < 0.05. ** indicates a *p*-value < 0.01. *** indicates a *p*-value < 0.001. Error bars indicate 95% confidence intervals.

The association between TILS effects on CCO activity and regional depth from the dorsal surface of the brain was assessed as a function of dorsoventral depth, treatment group classification, or both factors. Both Akaike information criterion (AIC) and Bayesian information criterion (BIC; Vrieze, 2012) were used for each possible model. The best-fitting model, with the lowest AIC and BIC, was one containing both dorsoventral depth and group classification factors. The resulting linear regression using this best-fit model is in Table 3. The estimated marginal means of each dorsoventral depth are represented in Figure 8. An R^2^ of 0.919 and an overall model value of p of <0.001 indicate that the variance in mean CCO activity at each dorsoventral depth can be explained by our explanatory variables, depth and treatment group. Comparisons of dorsoventral depth found no significant difference between 2 and 2.9 mm below the dorsal surface of the brain and 1–1.9 mm below the dorsal surface (*p* = 0.527). However, significant increases in CCO activity were found at 3–3.9 mm (*p* = 0.002), 4–4.9 mm (*p* = 0.002), and 6–6.9 mm (*p* < 0.001) below the dorsal surface of the brain, as well as a nonsignificant increase in activity at 5–5.9 mm below the surface (*p* = 0.155). In contrast, there was a significant decrease in CCO activity at 7–8.2 mm below the dorsal surface (*p* = 0.008).

**Table 3:** Linear regression of average cytochrome c oxidase activity (μmol of cytochrome c oxidized/min/g of wet tissue weight) with dorsoventral depth in mm and treatment group as factors.

Model fit measures						
			Overall model test			
Model	R	R^2^	F	df1	df2	*p*
1	0.959	0.919	22.8	9	18	< 0.001

Model Coefficients – Mean CCO activity (μmol of cytochrome c oxidized/min/g of wet tissue weight)				
Predictor	Estimate	SE	*t*	*p*
Intercept^a^	158.80	3.35	47.428	< 0.001
Depth in mm:				
2–2.9 – 1–1.9	−2.56	3.96	−0.645	0.527
3–3.9 – 1–1.9	14.26	3.96	3.599	0.002
4–4.9 – 1–1.9	14.17	3.96	3.576	0.002
5–5.9 – 1–1.9	5.89	3.96	1.486	0.155
6–6.9 – 1–1.9	32.87	3.96	8.296	< 0.001
7–8.2 – 1–1.9	−11.83	3.96	−2.987	0.008
Group:				
1 day – Sham	−3.54	2.99	−1.182	0.252
2 weeks – Sham	9.65	2.99	3.223	0.005
4 weeks – Sham	13.79	2.99	4.603	< 0.001

a Represents reference level.

### The dorsoventral biphasic CCO response to TILS does not parallel the decremental light distribution gradient in the brain

**Figure 9:**
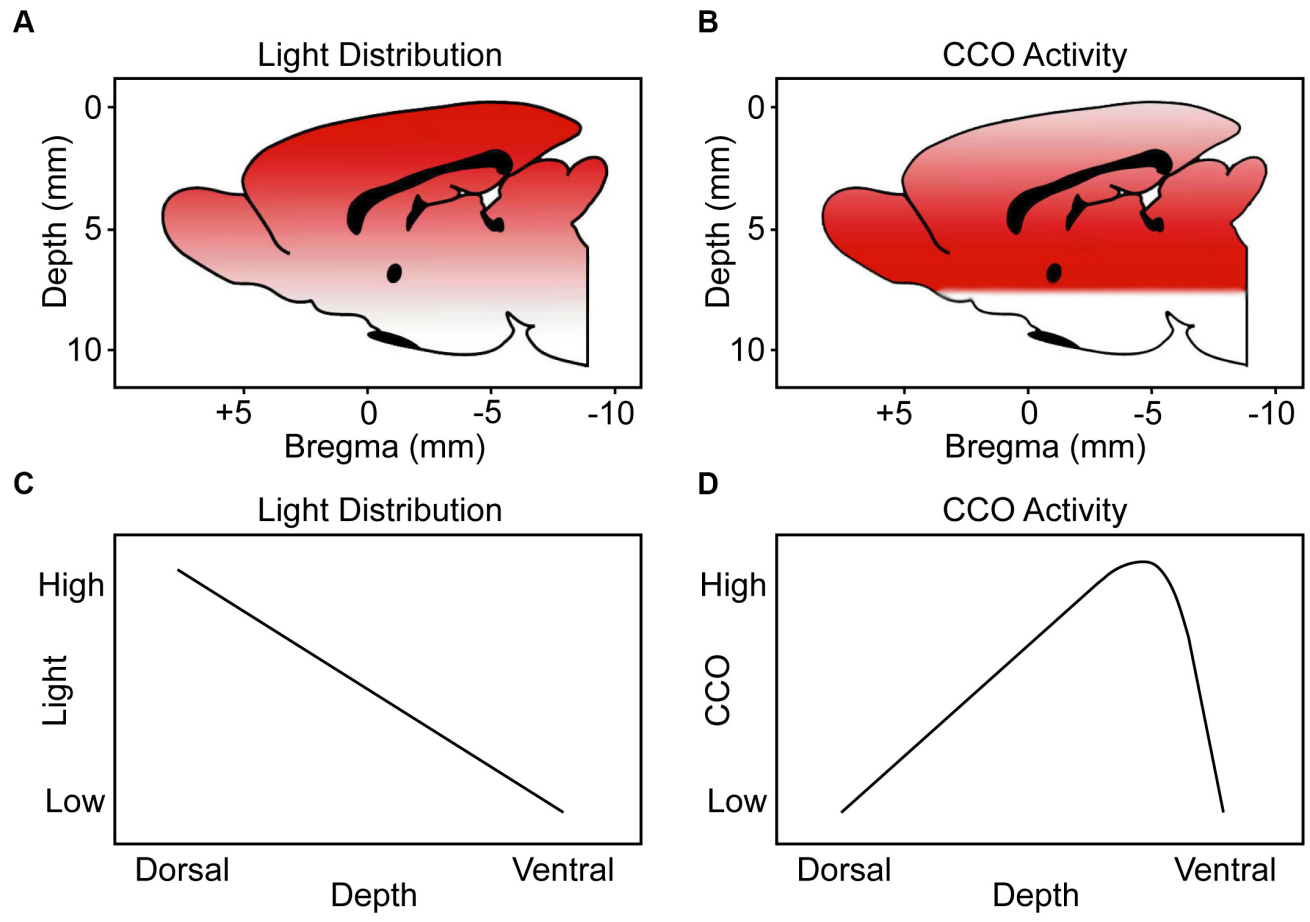
Schematic representation to visualize the light distribution and CCO activity through the dorsoventral depth (mm) of the brains of rats treated with TILS to the head surface. Our schematics are from a medial view of the rat brain (midsagittal plane) indicating the dorsoventral distance (depth from top brain surface) and anteroposterior distance (from the Bregma landmark on the skull) guided by coordinates verified with the stereotaxic atlas of Paxinos and Watson (2006). **(A)** Color gradient from red to white represents higher-to-lower level of light distribution. **(B)** Color gradient from red to white represents higher-to-lower CCO activity. **(C)** Linear function of light distribution along the dorsoventral depth. **(D)** Biphasic function of CCO activity changes in response to TILS. This figure illustrates that there is a biological hormetic dose–response of CCO activity to TILS, rather than simply a decreasing effect of light distribution from the dorsal to the ventral surfaces of the brain.

We measured in a freshly extracted brain from an additional rat that 8.5% of the irradiance applied at the dorsal brain surface reached the ventral surface. Figure 9 shows a stereotaxic brain schematic to visualize that light distribution along the dorsoventral depth (mm) of the rat brain after TILS to the head surface (Figure 9A) is different than the corresponding CCO activity changes (Figure 9B). While ventral surfaces receive less light dose, the decremental light distribution gradient (Figure 9C) does not correspond to the biphasic CCO response to TILS (Figure 9D). The biphasic biological response consisted of opposite effects to high and low light doses. Following a characteristic hormetic dose–response, CCO response increased as a function of decreasing light dose until reaching a peak response. After this point, a further decrease in light dose at the ventral surface caused CCO levels to drop below baseline (Figure 8). Therefore, these data are consistent with a true biological hormetic dose–response of CCO activity to light dose, rather than simply a decreasing light distribution from the dorsal to the ventral surfaces of the brain.

## Discussion

This sham-controlled study is the first to map the duration of action of *in vivo* PBM on cytochrome c oxidase (CCO). Our ANCOVA results demonstrated elevated CCO activity post-TILS in several brain ROIs. The infralimbic cortex displayed greater CCO activity at both 1-day and 2–4 weeks after TILS. This suggests a CCO upregulation in the infralimbic cortex after a single TILS treatment that appeared earlier than in other brain regions and that persisted for 2–4 weeks. Notably, the infralimbic cortex was the only cortical ROI to show a significant change in CCO activity after TILS, as well as the only ROI examined that exhibited a significant change 1 day after TILS. Interestingly, the infralimbic cortex is the brain region with the greatest increase in CCO activity after associative learning, suggesting that it may have a higher capacity for metabolic neuroplasticity (Gonzalez-Lima and Bruchey, 2004; Bruchey and Gonzalez-Lima, 2008; Rojas et al., 2012).

The neurobiological role of rat infralimbic cortex activity is of great behavioral significance for conditioned safety, as shown in many studies of fear extinction (Quirk et al., 2006). In particular, Kreutzmann et al. (2020) showed that temporary inactivation of the infralimbic cortex by muscimol during the behavioral fear expression test completely blocked the expression of conditioned safety. Furthermore, infralimbic cortex activity was required for the expression but not the acquisition of conditioned safety. This important study is consistent with our previous behavioral testing showing that improving CCO activity in the rat prefrontal cortex by photobiomodulation after fear extinction causes an improvement in the expression of conditioned safety tested 1 day later (Rojas et al., 2012). Furthermore, a single TILS session to the human prefrontal cortex causes significant reduction of fear in people with pathological fear (Zaizar et al., 2023).

The rat infralimbic cortex is understood to be homologous to the human prefrontal cortex (Ongür and Price, 2000), which is the brain region typically targeted in TILS studies of human subjects (Barrett and Gonzalez-Lima, 2013; Gonzalez-Lima and Barrett, 2014; Blanco et al., 2015, 2017; Vargas et al., 2017; Wang et al., 2017, 2018, 2019; Holmes et al., 2019; Pruitt et al., 2020; Saucedo et al., 2021). This mapping study suggests that CCO activity in prefrontal cortex is more impacted by a single TILS treatment than other brain regions. The prefrontal cortex is also unique among all other brain regions in that it receives highly processed information from all major forebrain systems (Miller et al., 2002).

Significant increases in CCO activity 2 weeks after TILS were also found in other brain regions. The lateral septum (LS) and accumbens core displayed a non-significant elevation of CCO activity at 1 day post-stimulation and a significant elevation in the 2-weeks post-TILS group. Since these two regions have subcortical neuroanatomic locations with greater dorsoventral depths compared to cortical regions, the LS and the accumbens core likely received somewhat less TILS light than cortical regions. Thus, it is possible that the LS and the accumbens core exhibited a delayed reaction to TILS. These regions are core components of the forebrain system associated with behaviorally rewarding functions, with major connections to the infralimbic cortex (Hoover and Vertes, 2007; Peters et al., 2009). Therefore, CCO increases in these regions may have followed in time as a result of the earlier metabolic neuroplasticity in the infralimbic cortex.

On the other hand, limbic regions in the hippocampus and amygdala showed opposite CCO effects to those seen in the forebrain reward system activated by TILS. Specifically, the CA3 sector and the molecular layer of the hippocampus exhibited a non-significant downward trend in CCO activity from sham in the 1-day post-TILS group. This CCO activity reduction demonstrated significance in the 2-weeks post-TILS group. The medial amygdala (AMe) showed a significant decrease in the 4-weeks group, but did not trend in either direction in the 1-day or 2-weeks post-TILS groups. These CCO decrements may also be related to the early TILS effect in the infralimbic cortex. There is consistent evidence in rat studies that increases in CCO and electrophysiological activity in the infralimbic cortex, such as after fear extinction learning, reduce the CCO and electrophysiological activity in the amygdala and hippocampus (Quirk et al., 2006). Interestingly, we recently found that a single TILS administration that upregulates prefrontal CCO (Wang et al., 2017) results in fear reduction in humans (Zaizar et al., 2023). Together, the various regional changes in CCO activity suggest that TILS produced long-lasting effects in forebrain networks associated with the infralimbic cortex.

We previously conducted the first detailed brain mapping study of chronic laser effects on brain regional CCO activity (Cardoso et al., 2022). Specifically, we used quantitative CCO histochemistry to map the differences in CCO activity of brain regions in healthy young (4 months old) and aged (20 months old) rats from control groups with sham stimulation and from treated groups with 58 consecutive days of transcranial laser PBM (810 nm wavelength and 100 mW power). We found that this daily PBM for nearly 2 months predominantly decreased regional brain CCO activity in the young rats, whereas it increased CCO activity in the older rats. Since aging predominantly decreased regional brain CCO activity in control rats, the chronic laser stimulation was beneficial for the older treated rats because it reversed aging-related CCO deficits. Therefore, the physiopathological context of this chronic laser treatment was aging-dependent, with young rats showing mainly inhibitory effects and older rats showing mainly stimulatory effects on CCO activity.

We further investigated brain network neuroplasticity caused by TILS using inter-regional correlation matrices of changes in CCO activity. This is a well-validated analytical method for evaluating functional connectivity in the brain of rats (Riha et al., 2011; Vélez-Hernández et al., 2014; Auchter et al., 2020; Cardoso et al., 2022). The most utility of this method is for evaluating overall patterns of modification in large brain networks rather than focusing on individual regions. The inter-regional correlation matrices revealed that the effects of laser stimulation at 1-day post-TILS resulted in 31 additional statistically significant pairwise partial correlations between ROIs as compared to the sham group. In these same ROIs, 23 significant partial correlations remained in the 2-weeks group compared to the sham group. Notably, 61 significant partial correlations were found in the 4-weeks group compared to the sham group. Generally, TILS increased the number of significant network correlations between brain ROIs as a function of time after administration. This suggests that TILS may augment functional connectivity between multiple networks in the brain. Changes in functional connectivity between some ROIs likely persist at least 4 weeks after stimulation.

Increases in inter-regional functional connectivity are consistent with the previous findings of Cardoso et al. (2022), which histochemically examined CCO activity effects of 58 consecutive days of TILS in young and aged rats. They discovered that chronic TILS increased systems-level correlativity activity in aged rats, such that functional connectivity of aged rats receiving TILS rose to a level similar to young control rats. They also found an increase in functional connectivity after TILS, which is consistent with our results.

We also evaluated the association between TILS effects on CCO activity and regional depth from the dorsal surface of the brain. Linear regression analysis of dorsoventral depth and CCO activity showed significant increases in CCO activity at 2–2.9 mm, 3–3.9 mm, 4–4.9 mm, and 6–6.9 mm, as well as a nonsignificant increase in CCO activity at 5–5.9 mm. There was also a significant decrease in CCO activity at 7–8.2 mm. The medial amygdala (AMe), the central amygdala (ACe), and the basolateral amygdala (AB) are located at the 7–8.2 mm depth. Therefore, these regions may have been exposed to less light than more dorsally located regions. However, that simple explanation could not account for the fact that the most dorsal regions showed no changes in CCO activity. Alternatively, we speculate that the observed decrease in CCO activity at this deeper dorsoventral depth was likely contributed by the top-down inhibitory network effect of the infralimbic cortex discussed earlier. In addition, model coefficients and estimated marginal means (pictured in Figure 8) support a biphasic (hormetic) association (Calabrese, 2008, 2015) of elevated mean CCO activity with increased depth from the dorsal surface of the brain, until a reduction at the 7–8.2 mm depth. Such opposite responses to lower and higher doses of PBM are characteristic of the hormetic dose–response function found in PBM experiments, both *in vitro* and *in vivo* (Huang et al., 2011). Our findings support the proposal of a biphasic, hormetic dose–response to TILS (Cardoso et al., 2022).

Our study reveals that a single administration of TILS appears to be sufficient to result in measurable changes in regional CCO activity and network functional connectivity. However, the main limitation of the present study is the small sample size, which may have prevented us from discovering more changes due to TILS. Due to the limited sample size, this study does not possess the statistical power to correct for multiple comparisons. A greater sample size would permit increased statistical power and could control for confounding variables. For example, desiccation of brain tissue as a result of prolonged freezing due to the COVID-19 pandemic damaged some brain sections, and one subject from the 4-weeks post-TILS group was excluded. Additionally, no measurements were recorded between 1 day and 2 weeks post-TILS. Thus, it is possible that other significant effects of TILS in cortical regions receiving more direct stimulation may result between 1-day and 2-weeks post-TILS.

In conclusion, we found statistically significant changes in CCO activity of brain ROIs at 1-day, 2-weeks, and 4-weeks post-TILS. The most notable significant CCO activity change after TILS recorded at 1-day post-TILS was in the infralimbic cortex, suggesting its earlier capacity for metabolic neuroplasticity. Other subcortical regions associated with infralimbic networks showed the greatest significant CCO changes at 2-weeks and 4-weeks post-TILS. It is also possible that ventral brain regions with less penetrance of TILS may demonstrate a delayed reaction in CCO activity to TILS. Inter-regional correlation analyses also support greater functional connectivity between brain ROIs after TILS, with increased significant pairwise activity correlations persisting for 4-weeks post-stimulation. The time course of CCO changes in activity and functional coupling indicate that different types of neuroenergetic plasticity may occur at different time scales after TILS, depending on the brain region and its depth from the cortex. The overall results are the first to demonstrate the duration of action of a single TILS session on CCO activity in the brain.

## Data availability statement

The raw data supporting the conclusions of this article will be made available by the authors, without undue reservation.

## Ethics statement

The animal study was approved by The University of Texas at Austin, Institutional Animal Care and Use Committee. The study was conducted in accordance with the local legislation and institutional requirements.

## Author contributions

FG-L and DB designed the experiment and performed the TILS treatment. DB performed the live animal work, including handling, decapitation, brain extraction, and freezing the tissue. ZW sectioned the brains, histochemically stained the tissue, and gathered the brain metabolic data via optical densitometry, under supervision by FG-L and RD. ZW and DB performed the statistical analysis. FG-L, DB, and ZW interpreted the results and wrote the paper. RD, AN, and SV assisted in histochemical staining, sectioning, and optical densitometry. All authors contributed to the article and approved the submitted version.

## Conflict of interest

The authors declare that the research was conducted in the absence of any commercial or financial relationships that could be construed as a potential conflict of interest.

## Publisher’s note

All claims expressed in this article are solely those of the authors and do not necessarily represent those of their affiliated organizations, or those of the publisher, the editors and the reviewers. Any product that may be evaluated in this article, or claim that may be made by its manufacturer, is not guaranteed or endorsed by the publisher.
